# Assessing the performance of different approaches for functional and taxonomic annotation of metagenomes

**DOI:** 10.1186/s12864-019-6289-6

**Published:** 2019-12-10

**Authors:** Javier Tamames, Marta Cobo-Simón, Fernando Puente-Sánchez

**Affiliations:** Systems Biology Department, Centro Nacional de Biotecnología, CSIC, C/Darwin 3, 28049 Madrid, Spain

**Keywords:** Metagenomics, Functional annotation, Taxonomic annotation, Assembly

## Abstract

**Background:**

Metagenomes can be analysed using different approaches and tools. One of the most important distinctions is the way to perform taxonomic and functional assignment, choosing between the use of assembly algorithms or the direct analysis of raw sequence reads instead by homology searching, k-mer analysys, or detection of marker genes. Many instances of each approach can be found in the literature, but to the best of our knowledge no evaluation of their different performances has been carried on, and we question if their results are comparable.

**Results:**

We have analysed several real and mock metagenomes using different methodologies and tools, and compared the resulting taxonomic and functional profiles. Our results show that database completeness (the representation of diverse organisms and taxa in it) is the main factor determining the performance of the methods relying on direct read assignment either by homology, k-mer composition or similarity to marker genes, while methods relying on assembly and assignment of predicted genes are most influenced by metagenomic size, that in turn determines the completeness of the assembly (the percentage of read that were assembled).

**Conclusions:**

Although differences exist, taxonomic profiles are rather similar between raw read assignment and assembly assignment methods, while they are more divergent for methods based on k-mers and marker genes. Regarding functional annotation, analysis of raw reads retrieves more functions, but it also makes a substantial number of over-predictions. Assembly methods are more advantageous as the size of the metagenome grows bigger.

## Background

Since its beginnings in the early 2000s, metagenomics has emerged as a very powerful way to assess the functional and taxonomic composition of microbiomes. The improvement in high-throughput sequencing technologies, computational power and bioinformatic methods have made metagenomics affordable and attainable, increasingly becoming a routine methodology for many laboratories.

The usual goal of metagenomics is to provide functional and taxonomic profiles of the microbiome, that is, to know the abundances of taxa and functions. A metagenomic experiment consists of a first wet-lab part, where DNA from samples is extracted and sequenced, and a second in silico part, where bioinformatics analysis of the sequences is carried out. There is not a golden standard for performing metagenomic experiments, especially regarding the bioinformatics used for the analysis.

Usually, one of the first steps in the analysis involves the assembly of the raw metagenomic reads after quality filtering. The objective is to obtain contigs, where genes can be predicted and then annotated, usually by means of comparisons against reference databases. It is sensible to think that the taxonomic and functional identification is more precise having the full gene than just the fragment of it contained in a short read. Also, taxonomic classification benefits of having contiguous genes, because since they come from the same genome, non-annotated genes can be ascribed to the taxon of their neighbouring genes. Therefore, obtaining an assembly can facilitate considerably the subsequent annotation steps.

However, de novo metagenomic assembly is a complex task: the performance of the assembly is dependent on the number of sequences and the diversity of the microbiome (richness and evenness of the present species) [1], and a fraction of reads will always remain unassembled. Microbiomes of high diversity or high richness (those presenting many different species) such as those of soils, are harder to assemble, likely to produce more misassembles and chimerism [2], and will produce smaller contigs.

From a computational point of view, the assembly step often requires large resources, especially in terms of memory usage, although modern assemblers have somewhat reduced this constraint. Different assemblers are available, which use diverse algorithms and heuristics and hence may produce different results, whose assessment is difficult.

Probably because of these problems, some authors prefer to skip the assembly step and proceed to the direct functional/taxonomic annotation of the raw reads, especially when the aim is just to obtain a functional or taxonomic profile of the metagenome [3–8]. This approach provides counts for the abundance of taxa and functions based on the similarity of the raw reads to corresponding genes in the database. There are two main drawbacks of working with raw reads in this way: first, since it is based on homology searches for millions of sequences against huge reference databases, it usually needs large CPU usage, especially taking into account that for taxonomic assignment the reference database must be as complete as possible to minimize errors [9]; and second, the sequences could be too short to produce accurate assignments [10, 11]. Also, it is generally harder to annotate functions than taxa, because short reads are often not discriminative enough to distinguish between functions, since they may map to promiscuous domains that can be shared between very different protein.

Another alternative to assembly is to count the k-mer frequency of the raw reads, and compare it to a model trained with sequences from known genomes, as implemented in Kraken2 [12] or Centrifuge [13]. As k-mer usage is linked to the phylogeny and not to function, these methods can be used only for taxonomic assignment.

Finally, also for taxonomic profiling other methods rely on the identification of phylogenetic marker genes in raw reads to estimate the abundance of each taxa in the metagenome, for instance Metaphlan2 [14] or TIPP [15]. These methods must be considered profilers, since they do not attempt to classify the full set of reads, but instead recognize the identity of particular marker genes to infer community composition from these.

These different methods (assemblies, raw reads, k-mer composition and marker gene profiling) are likely to produce different results. While benchmarking and comparison of metagenomic software has been extensively done, for instance in the GAGE (Critical evaluation of genome and metagenome assemblies) [16] and CAMI (Critical Assessment of Metagenome Interpretation) [17] exercises, the influence of these different annotation strategies has been less studied. We have scarce information on how diverse the results of these approaches are, and whether they are so different as to compromise the subsequent biological interpretation of the data. This is a relevant point, since these methods are being used indistinctly for metagenomic analyses and their results could not be comparable if the differences are large.

The objective of the present analysis is to estimate the differences between all these approaches. To this end, we will functionally and taxonomically classify several real and mock metagenomes using direct assignment of the raw reads, or assembling the metagenomes first, annotating the genes, and then annotating the reads using their mapping to the genes [18, 19]. For taxonomic analysis, we also use Kraken2 as a k-mer classifier, and Metaphlan2 as a marker gene classifier.

The mock communities of known composition can help us to evaluate the goodness of the results. Even if mock communities are rather less complex than real ones, they are valuable tools for having a framework to compare the annotations done by different methods to the real expectations.

We aim to illustrate how different approaches can lead to diverse results, and therefore different interpretations of the underlying biological reality. We hope that this can help in the informed choice of the most adequate method according to the particular characteristics of the dataset.

## Results

### Mock communities

To better estimate the performances of each method of assignments, we created mock communities simulating microbiomes of marine, thermal, and gut environments. We selected 35 complete genomes from species known to be associated to these environments, according to a compiled list of preferences between taxa and habitats [20], and created mock metagenomes by selecting a variable number (from 0.2 M to 5 M) of reads from them, in diverse proportions. The composition of these mock metagenomes can be found in Additional file 8: Table S1.

#### Taxonomic annotations

**Fig. 1:**
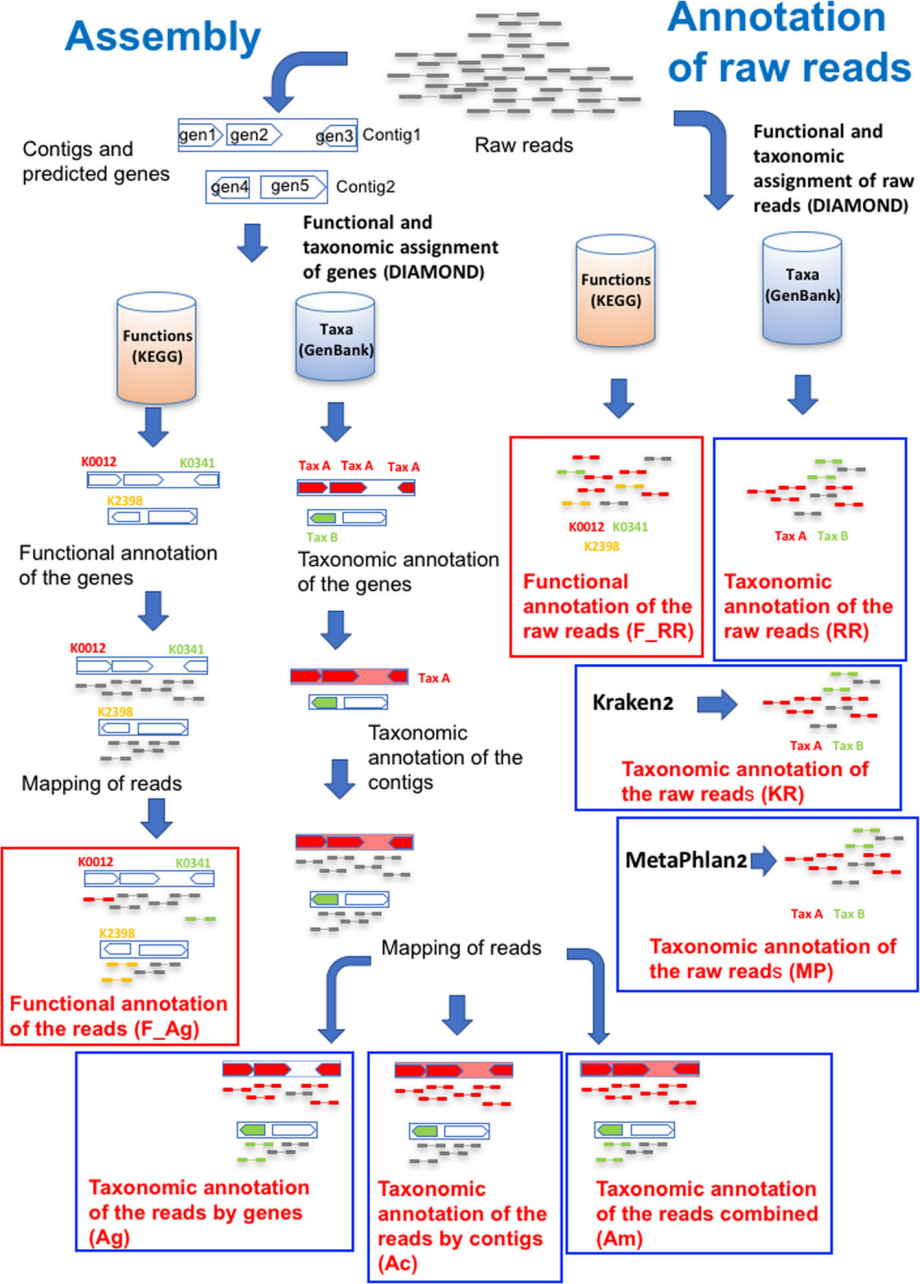
Schematic description of the procedure followed for the analysis. Boxed in blue, taxonomic annotations. In red, functional (KEGG) annotations

We used different methods to taxonomically assign the reads from these metagenomes (see Fig. 1 and methods for full details): 1) We ran a homology search of the reads against the GenBank NR database, followed by assignment using the last common ancestor (LCA) of the hits. We termed this approach “assignment to raw reads” (RR). 2) We also used the SqueezeMeta software [21] to proceed with a standard metagenomic analysis pipeline: assembly of the genomes using Megahit [18], prediction of genes using Prodigal [22], taxonomic assignment of these genes by homology search against the GenBank nr database (followed by LCA assignment as above), taxonomic assignment of the contig to the consensus taxon of its constituent genes, mapping of the reads to the contigs using Bowtie2, and taxonomic annotation of the reads according to the taxon of the gene (assembly by genes, Ag) or contig (assembly by contigs, Ac) they mapped to. We also used a combined approach in which the read inherited the annotation of the contig in first place, or the one for the gene if the contig was not annotated (assembly combined, Am). 3) In addition, we used Kraken2, a k-mer profiler that assigns reads to the most likely taxon by compositional similarity. 4) Finally, we used Metaphlan2, which attempts to find reads corresponding to clade-specific genes to assign the corresponding read to the target clade.

**Fig. 2:**
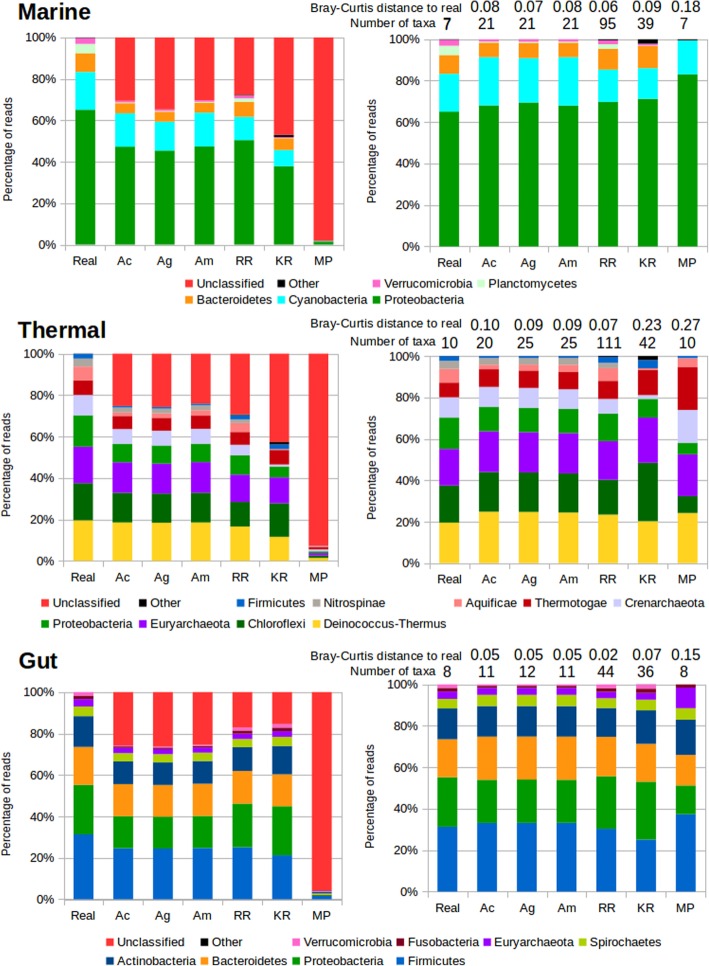
Taxonomic assignments for the mock metagenomes. Left panels show the results for all the reads, right panels show the results removing unclassified reads and scaling to 100%. Real: Real composition of the mock community. Ac, Assembly and mapping reads to contigs. Ag, Same but mapping reads to genes. Am, same but mapping genes first to contigs, then to genes. RR, raw reads assignment. KR: Kraken2. MP: Metaphlan2. Numbers above the bars in the right panels correspond to the Bray-Curtis distance to the composition of the original microbiome, and the number of taxa (phyla) recovered by each method, with the real number of taxa present in the mock metagenome indicated in the “Real” column

We first will focus in the 1 M dataset for discussing the results. The results for the phylum rank can be seen in Fig. 2, and for the family rank in Additional file 1: Figure S1.

The methods classifying more reads are RR for the marine mock metagenome, Am for the thermal, and Kraken2 for the gut. As expected, the assembly approaches work better when the assemblies recruit more reads (the percentage of mapped reads in the assemblies is 75, 84 and 81% for marine, thermal and gut, respectively). Kraken2 seems to be especially suited to classify gut metagenomes, but misses many reads for metagenomes from other environments. RR also classifies more reads for gut metagenomes, indicating that the representation of related genomes and species in the database, which is higher for gut genomes, is an important factor. We measured the Bray-Curtis dissimilarities to the real taxonomic composition of the mock metagenome to evaluate the closeness of the observed results to the expected ones. The results are rather close to the original composition for the assembly approaches and RR, with best results for the gut metagenome. Kraken2 performs well for the marine and gut metagenomes, even if it misses entire phyla in some instances (for example, Nitrospinae in the thermal metagenome). Metaphlan2 provides the more distant profile in all cases. The Bray-Curtis dissimilarities between the taxonomic profiles generated by each method can be seen in Additional file 2: Figure S2. The RR and assembly approaches, which relied on homology annotations, led to similar results. On the other hand, the results from Kraken2 and Metaphlan2 were markedly different from the others.

We also inspected the number of reported phyla by each method. Excess of predicted phyla will be produced by incorrect assignments. Metaphlan2 is the only method that reports the exact number of phyla in all the mock microbiomes, while the assembly approaches provide a few more, and RR and Kraken2 report a higher number of superfluous taxa. Especially RR produces a very inflated number (more than ten times higher for the thermal mock microbiome). The version of Kraken2 that we used provided a maximum of 42 phyla for training, and therefore this is the maximum number of phyla that it will predict. In all cases the number is close to this top, indicating that Kraken2 predicts almost all taxa it has in its training set, irrespectively of the environment.

**Fig. 3:**
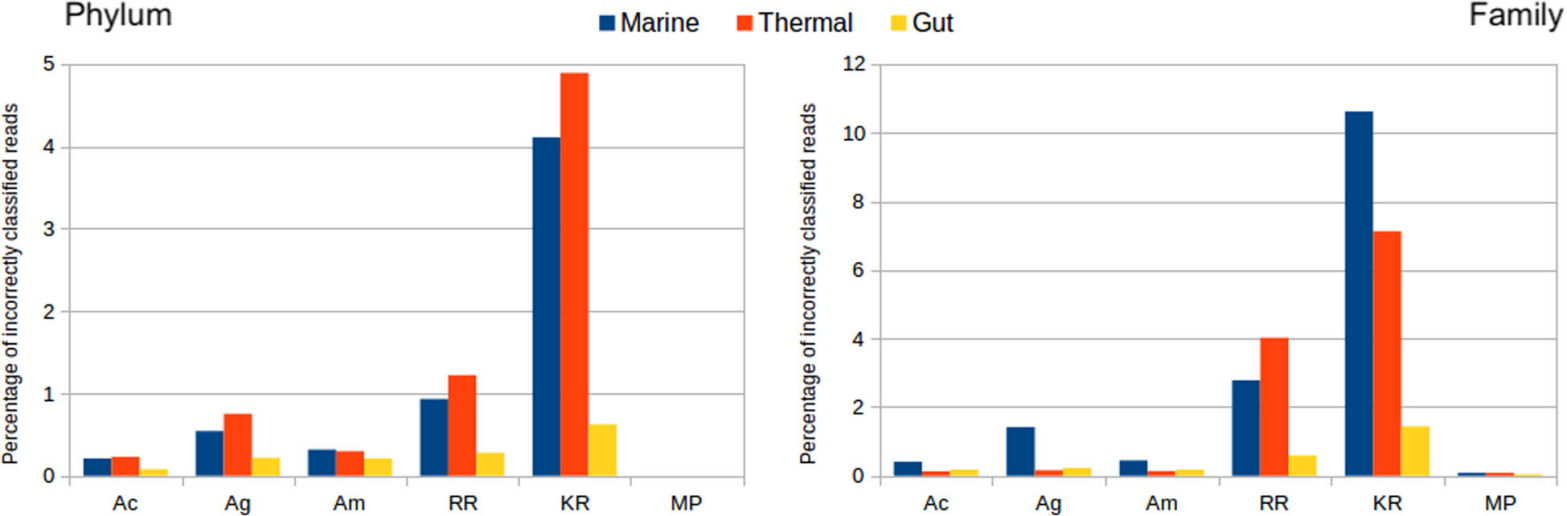
Percentage of discordant assignments between the different methods, for mock metagenomes. Only reads that were classified by both compared methods are considered (i.e. unclassified reads by either method are excluded). A: Assignment by Megahit assembly mapping to: (g: genes; c: contigs; m: combination of contigs and genes). RR: Assignment by raw reads; KR: Kraken2; MP: Metaphlan2

We next measured the error by inspecting the accuracy of the taxonomic annotations of the reads using the different methods (Fig. 3). All methods perform well (less that 1% error) for the gut metagenome at the phylum rank, and also at the family rank. Nevertheless, substantial differences appear for the other two environments, where errors increase notably. At phylum rank, more errors are done for the thermal metagenome, while at family rank, the marine metagenome is the most challenging. This is unrelated to the number of taxa in both metagenomes, as the thermal set has both more phyla and families. The most precise method is Metaphlan2, that makes no errors, although the low number of reads classified with this method produces a skewed composition as seen in Fig. 2. The assembly methods have less that 1% error in all cases, and annotation by contigs is more accurate than by genes, evidencing the advantage of having contextual information. RR taxonomic annotation exceeds the error rate of the assemblies, reaching 4% for the thermal metagenome at the family level. Kraken2 is the method making more errors, more than 4% for thermal and marine metagenomes at the phylum level, and reaching more than 10% for the marine metagenome at the family level. This is also reflected in the high amount of “Other taxa” classifications for Kraken2 in the Fig. 2.

The results were almost identical when replacing the megahit assembler by metaSPAdes [23], as it can be seen by the very low Bray-Curtis dissimilarities between Megahit and metaSPAdes results (Additional file 3: Figure S3).

**Fig. 4:**
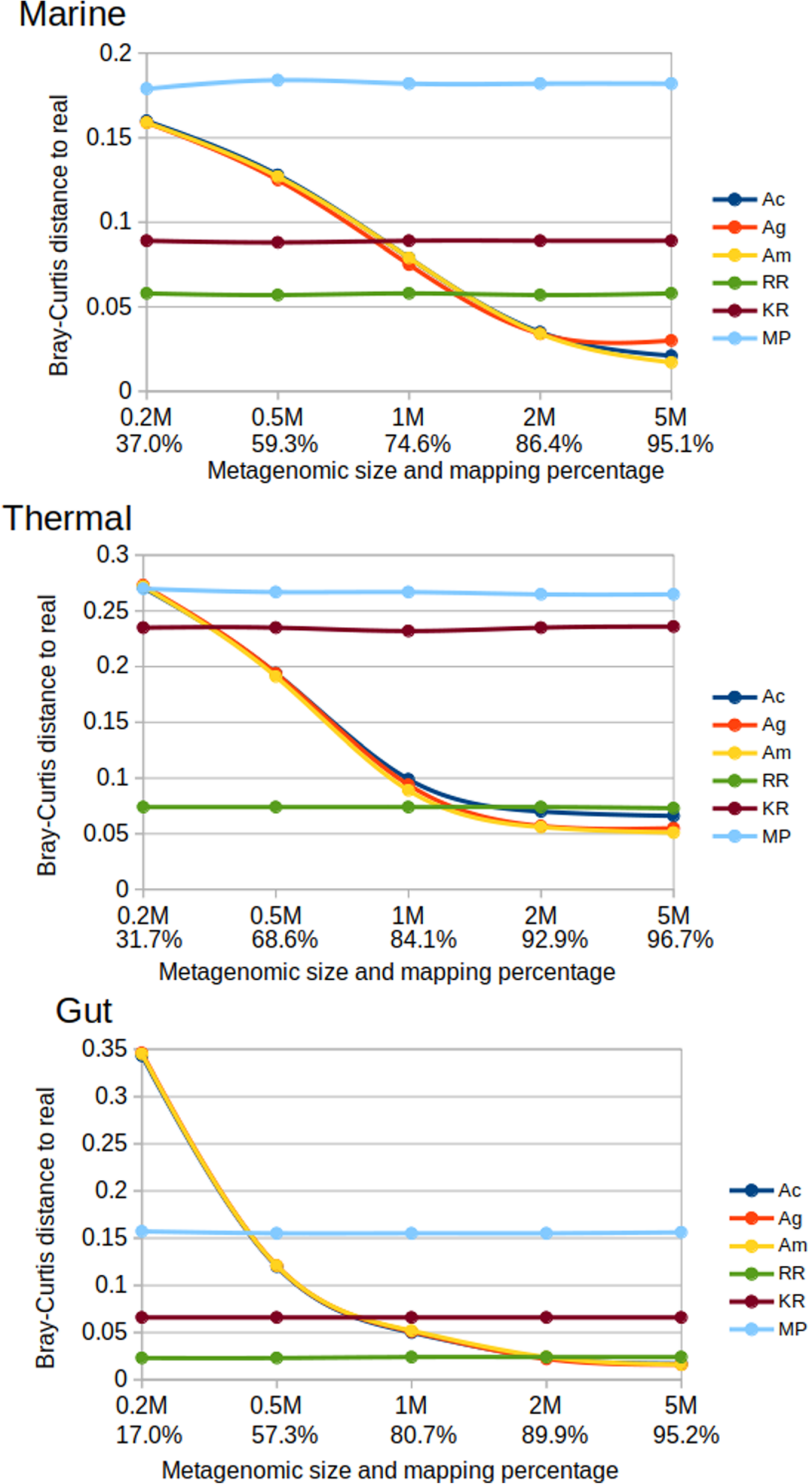
Bray-Curtis distance to the real composition of the mock metagenomes. For several sample sizes, at phylum rank. Ac, Assembly and mapping reads to contigs. Ag, Same but mapping reads to genes. Am, same but mapping genes first to contigs, then to genes. RR, raw reads assignment. KR: Kraken2. MP: Metaphlan2

We were aware that our results could be dependent on metagenomic size, especially those related to the assemblies for which the number of sequences is a critical factor. Therefore, we did additional tests to evaluate the performance of each method regarding metagenomic size. Our hypothesis was that methods that classify reads independently (RR, Kraken2 and Metaphlan2) would not be influenced, while the annotation by assembly could be seriously impacted. We created several mock metagenomes of different sizes for marine, thermal and gut environments, extracting reads from genomes strongly associated with these environments [20]. We created mock metagenomes for 200.000 (0.2 M), 500.000 (0.5 M), 1.000.000 (1 M), 2.000.000 (2 M) and 5.000.000 (5 M) paired sequences, all with the same composition of species (Additional file 8: Table S1). We annotated these datasets using the different methods, and calculated the Bray-Curtis distance between the resulting distribution of taxa and the real one. The results can be seen in Fig. 4 for the phylum rank, and in Additional file 4: Figure S4 for the family rank.

As we expected, RR, Kraken2 and Metaphlan2 are not affected by the size of the metagenome. Metaphlan2 is the method diverging more from the actual composition, except for the thermal mock community at family rank. Of these three methods directly assigning reads, RR is clearly the one providing the closest estimation to the real composition. Again, these methods perform much better for the gut mock metagenome than for the rest.

The assembly methods are, as expected, highly dependent of the amount of reads that can be assembled. For very small samples, where less than 50% of the reads are mapped to the assembly, it provides much more divergent classifications than other methods. When the percentage of assembled reads is in the range of 80–85%, they obtain similar results than RR. When the percentage of assembled reads is higher than that, taxonomic annotation by assembly outperforms the other methods. This indicates that the coverage of the metagenome (the number of times that each base was sequenced), which is directly related to the percentage of assembled reads, can be seen as the factor determining if it is more advantageous using RR or assembly methods for analysing metagenomes.

#### Functional annotations

**Fig. 5:**
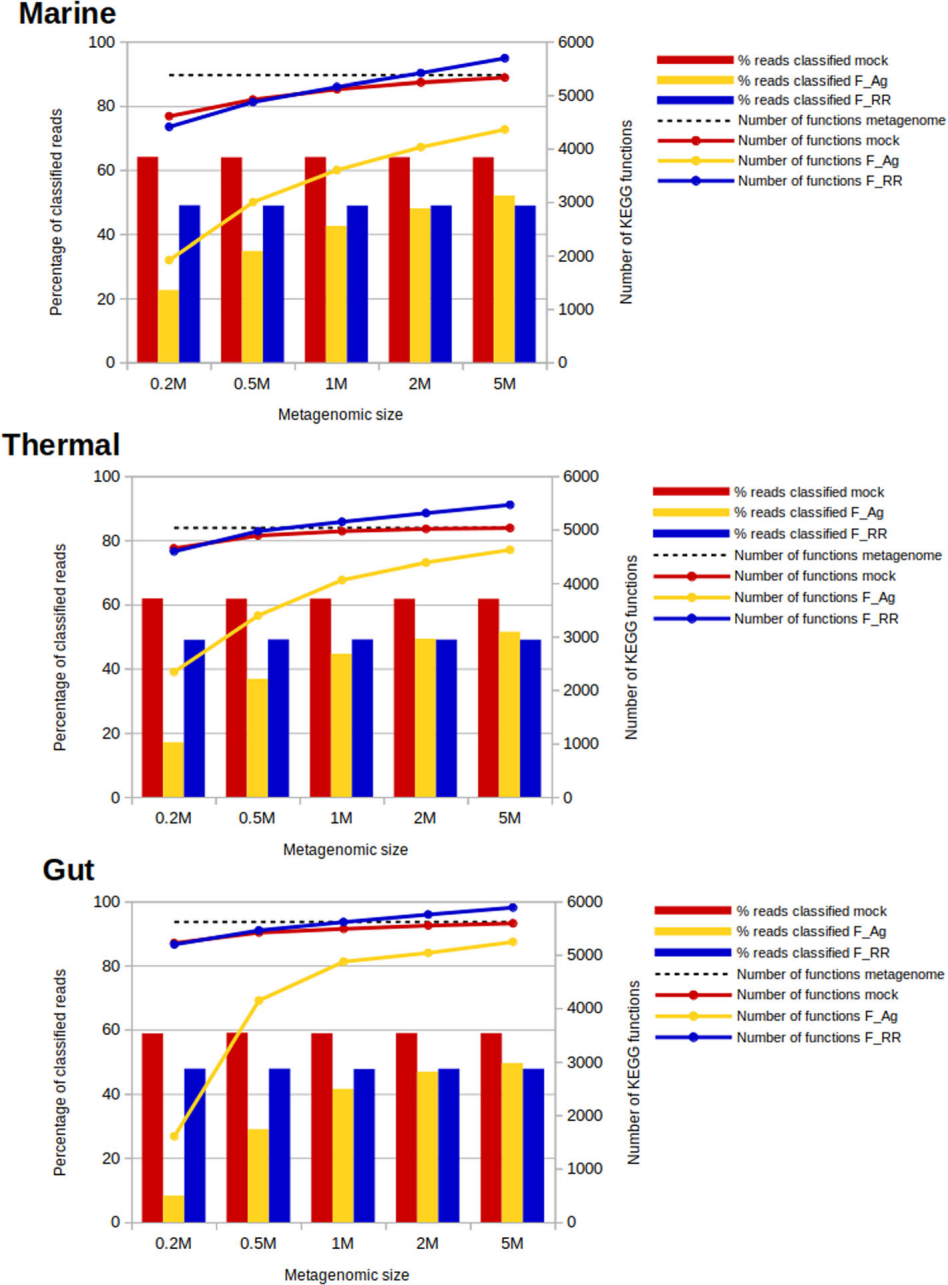
Functional classification of the mock metagenomes. Percentage of reads classified to KEGG functions (bars) and number of KEGG functions (lines) provided by the different functional assignment methods, for the different sizes of the mock metagenomes. The data labelled as “mock” correspond to the annotations of the reads based on the original annotations of the genomes. The data corresponding to “metagenome” correspond to the total number of functions in all the genomes used to build the mock communities

We also analysed the functional assignment for these mock metagenomes. The reference was the annotation of genes to KEGG functions. We classified the reads using the Assembly (F_Ag) and Raw Read (F_RR) annotation approaches. Kraken2 and Metaphlan2 were skipped since they do not provide functional annotation, and Ac and Am because there is not a contig annotation for functions (each gene has a different function). The results can be seen in the Fig. 5.

The maximum percentage of reads that can be functionally classified is around 60% for all metagenomes, the ones mapping to functionally annotated genes in the reference genomes. The rest correspond to reads from genes with no known function or with no associated KEGG. RR classification classifies around 50% of the reads in all cases. The variation with metagenomic size (the number of picked reads) is almost inexistent because the reads are extracted from the same background distribution of functions and they are annotated independently. F_Ag functional assignment, in turn, varies with size since it depends on metagenomic coverage, as stated above. We can see that for the biggest size (5 M), the percentage of assignments is larger for F_Ag than for F_RR. In this case there are no evident differences regarding the diverse environments.

Concerning the number of functions detected, it can be seen how the F_RR approach is over-predicting the number of functions, exceeding these actually present in the complete metagenome. This is an indication that this method is producing false positives, and the number of predicted functions increases linearly and shows no saturation, in contrast to the real number of functions. On the other hand, F_Ag produces a very low number of functions when the metagenomes are small, but it quickly increases to numbers close to the real ones for bigger sizes.

**Fig. 6:**
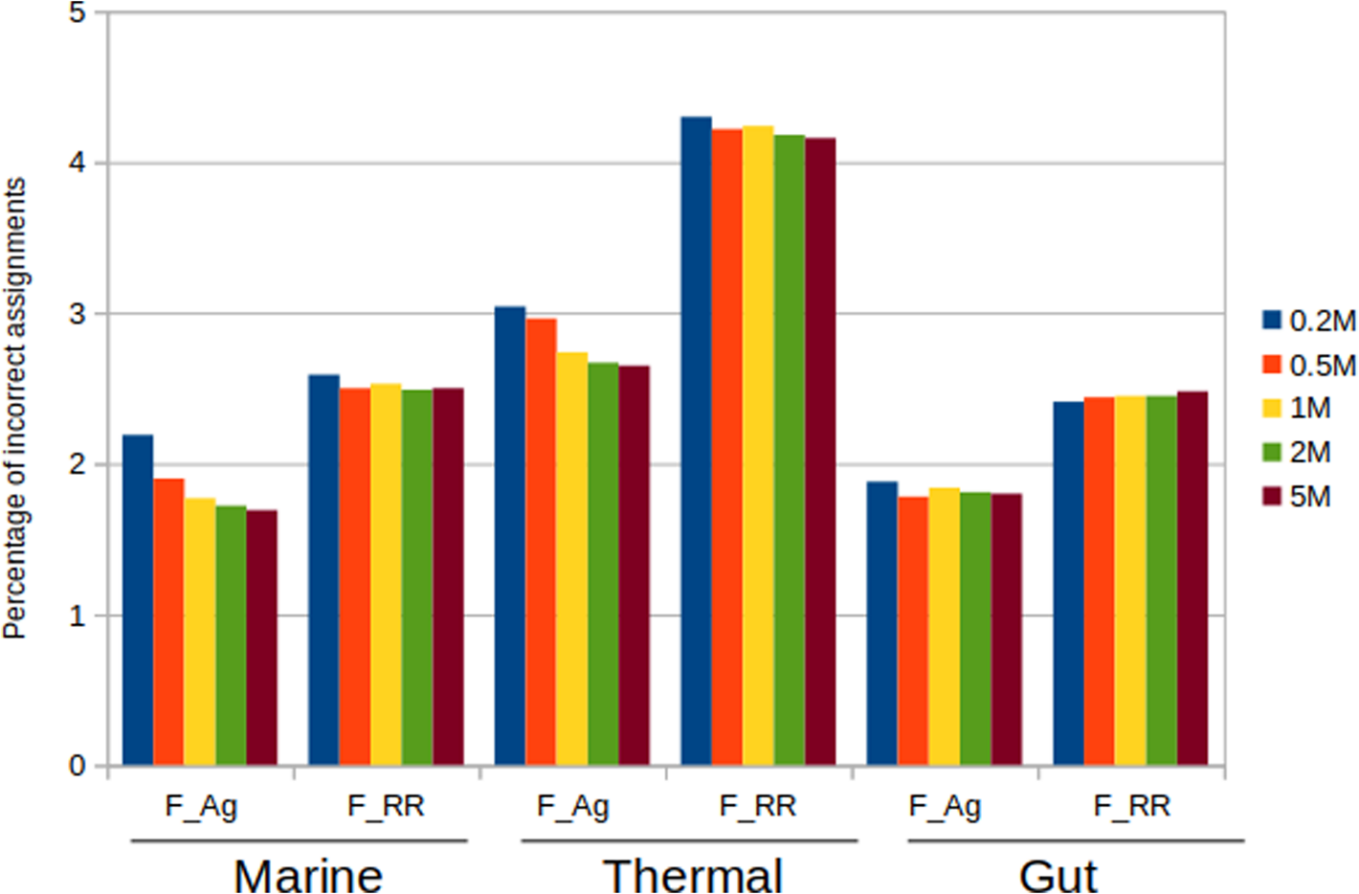
Incorrect functional assignments for the mock metagenomes. Shown as a percentage of the total number of reads. A read is incorrectly annotated when the provided function is different than the one from the original gene in the genome

We also quantified the number of wrong annotations by comparing the functional annotation of reads by each method with regard to the real scenario. The results can be seen in Fig. 6, and show that F_Ag has consistently a lower number of errors than F_RR, for all data sets. The differences between methods (discordant annotations) can also be seen in Additional file 9: Table S2.

F_RR assignments are always more error-prone. As for the taxonomic analysis, the thermal metagenome is the most difficult to annotate, and the gut one the easiest. The percentage of errors does not vary with sizes, and it is above 4% in the thermal metagenome. The F_Ag annotations are more precise, not exceeding the threshold of 3% errors. The influence of sizes can be noticed also here, with usually fewer errors in the bigger metagenomic sizes, but this trend is not so marked as for taxonomic annotations. For instance, the gut example shows a very stable error rate around 1.8%, irrespectively of the metagenomic size.

### Real metagenomes

Using methods described above, we analysed three different metagenomes coming from different environments, coincident with the mock communities studied previously: a thermal microbial mat metagenome from a hot spring in Huinay (Chile) [24], a marine sample from the Malaspina expedition [25], and a gut metagenome from the Human Microbiome Project [26] (thermal, marine and gut from now on).

#### Taxonomic annotations

**Fig. 7:**
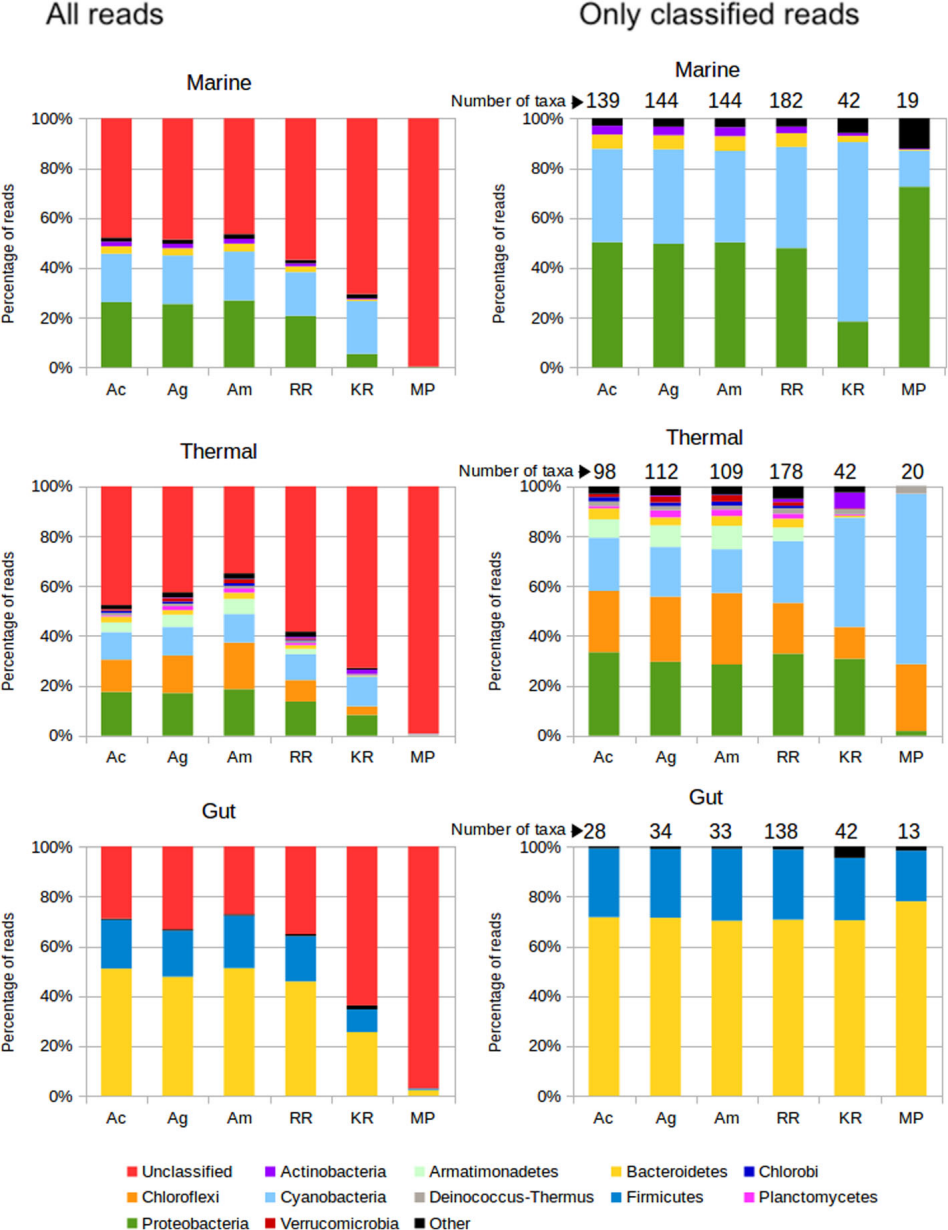
Comparison of taxonomic assignments by different methods, for real metagenomes. Ac, Assembly and mapping reads to contigs. Ag, Same but mapping reads to genes. Am, same but mapping genes first to contigs, then to genes. RR, raw reads assignment. KR: Kraken. MP: Metaphlan2. Left: All reads considered. Right: Discounting unclassified reads. These panels also show the number of obtained phyla

The results of the taxonomic annotation can be seen in Fig. 7, for the assignments at phylum rank. The results at family taxonomic rank are shown in Additional file 5: Figure S5, and show similar trends. The Bray-Curtis dissimilarities between the profiles generated by each method can be seen in Additional file 6: Figure S6.

Assembly methods achieve the highest number of classified reads in the three metagenomes. According to the results for mock communities, we anticipated that the amount of classifications by these methods would be related to the percentage of reads that were assembled. This will be ultimately related to the total size of the metagenome and the diversity of the community. The ratio of mapped reads is 72, 93, and 94% for the marine, thermal and gut samples, respectively. These numbers set the maximum percentage of reads that can be assigned by the assembly approaches. The most complete classification is achieved for the gut sample, allowing the taxonomic assignment of 73% of the reads. A substantial reduction is observed for the thermal sample (65% of reads assigned), even if the percentage of mapped reads is almost the same. This must be attributed to representation biases in the reference databases. This sample belongs to a much less studied habitat, and therefore the corresponding taxa are expected to be less represented in the databases, complicating the assignment. Finally, the marine sample is the most difficult to annotate by assembly (51% of the reads), because of the lower percentage of assembled reads.

The percentage of assignment is higher when using the combination of mapping reads to genes and contigs (Ac). Using the contig annotation can overcome unannotated genes, while gene annotations are not affected by the lack of consensus needed for contig assignment.

Taxonomic annotation of the raw reads (RR) resulted in 10–20% less classified reads, with the gut metagenome being the best annotated (65%), and marine and thermal having similar percentages (42–45% annotated reads). Kraken2 classification provides less annotations, around 25–30% less than the assembly. Again, the gut metagenome is the one having more assignments, benefiting of the increased completeness of the databases in gut-associated taxa. Finally, Metaphlan2 is able to classify very few reads, which is expected because it only annotates marker (clade-specific) genes.

The relative taxonomic composition at the phylum level obtained by each approach, discounting the effect of the unclassified reads, can be also seen in Fig. 7. Ideally, we should expect the same composition for all methods for the same metagenome, but instead we see that they diverge substantially. One of the most affected phylum is Cyanobacteria, present in thermal and marine samples. Assembly approaches report lower quantities for this taxon than RR and especially Kraken2, which greatly increases its proportion in the two datasets to unrealistic values, particularly in the case of the marine sample. The gene marker approach of MetaPhlan2 predicts less Cyanobacteria than the rest in the marine sample, but much more than the others in the thermal sample. The rest of the taxa are affected in different ways. Rare taxa such as Armatimonadetes in the thermal sample are obtained in greater abundance by the assembly approaches, and are ignored by Kraken2 and Methaphlan2, probably because of the absence of complete genomes belonging to these phyla in their training data sets. This is an example of how the gaps in the representation of taxa in the set of available complete genomes can hamper the taxonomic annotations of methods based on them [9, 27].

While the inferred composition of the gut metagenome is roughly the same for all approaches, the marine and thermal metagenomes vary slightly between raw reads and assembly, and greatly for Kraken2 and Metaphlan2. These divergences can be seen in the Bray-Curtis dissimilarity values in Additional file 6: Figure S6. The thermal metagenome shows big variations that affect for instance the determination of the most abundant taxon in the sample (Chloroflexi by assembly, Proteobacteria by raw reads, and Cyanobacteria by Kraken2 and Metaphlan2). Therefore, the choice of the method can influence greatly the ecological inferences obtained from the analysis.

**Fig. 8:**
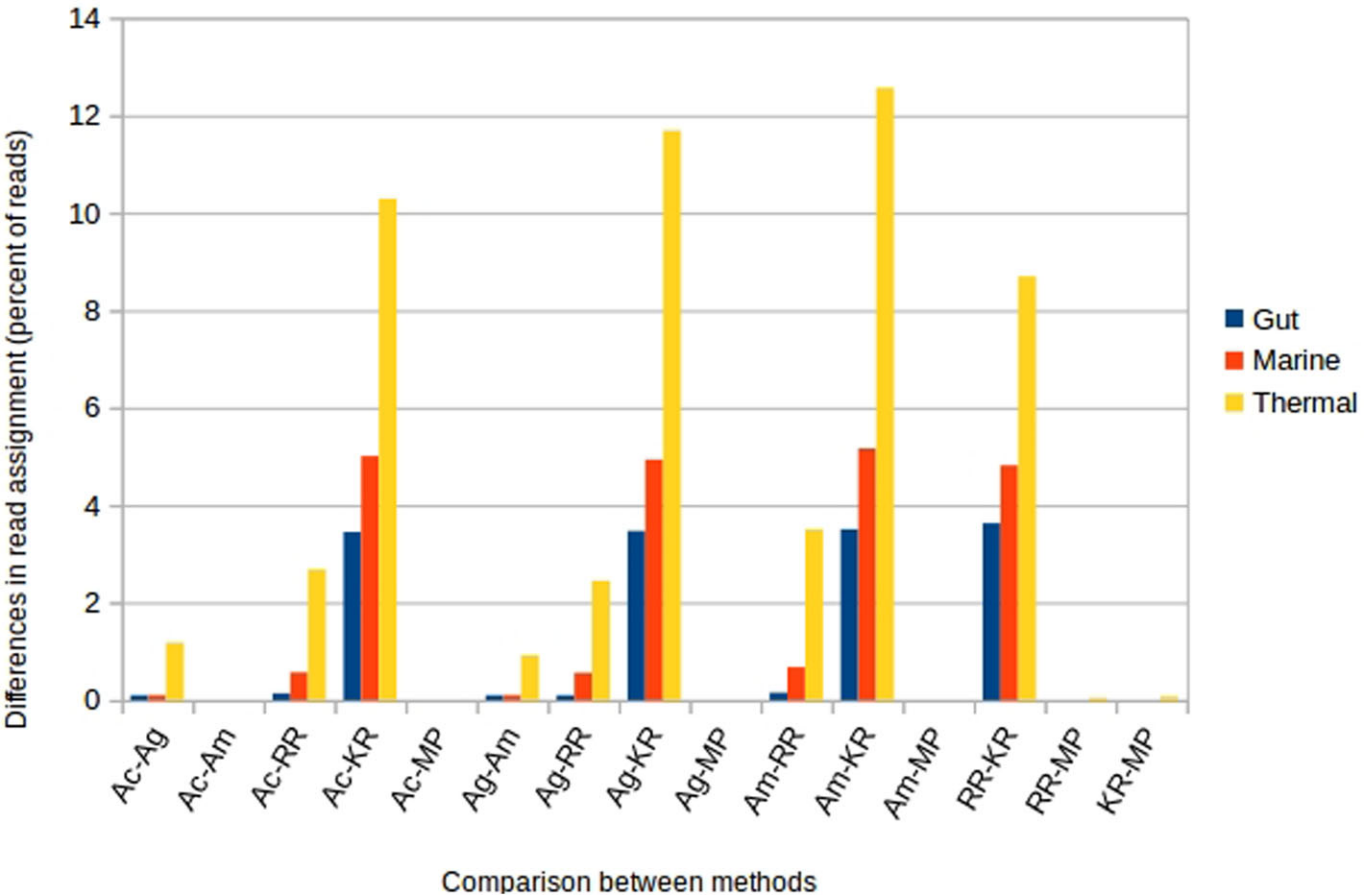
Percentage of incorrect taxonomic assigned reads, for real metagenomes. Left panel, phylum taxonomic rank. Right panel, family taxonomic rank. Ac, Megahit assembly and mapping reads to contigs. Ag, Same but mapping reads to genes. Am, same but mapping genes first to contigs, then to genes. RR, raw reads assignment. KR: Kraken. MP: Metaphlan2

We compared the discrepancies between the assignments done by different methods, by counting the cases in which the annotations were different at the phylum level (not-annotated reads were not considered). The results can be seen in Fig. 8. Consistently with the previous results, the thermal dataset is the one having more differences. The differences between the assembly methods are very low, and are also low between RR and assembly methods (less than 3% of the reads in the thermal dataset, less than 1% in the others). On the contrary, the differences were much bigger between Kraken2 and the rest: more than 8% for the thermal dataset, more than 4% for the marine, and almost 4% for the gut. This indicates again that the Kraken2 assignment is more dissimilar than the rest.

The statistical significances of the differences between different methods are shown in Additional file 7: Figure S7. The three assembly-based methods and RR were significantly (Kruskal-Wallis *p* < 0.05) more similar to the rest, while Kraken2 and Metaphlan2 were significantly (Kruskal-Wallis *p* < 0.05) more dissimilar to other methods.

#### Functional annotations

**Fig. 9:**
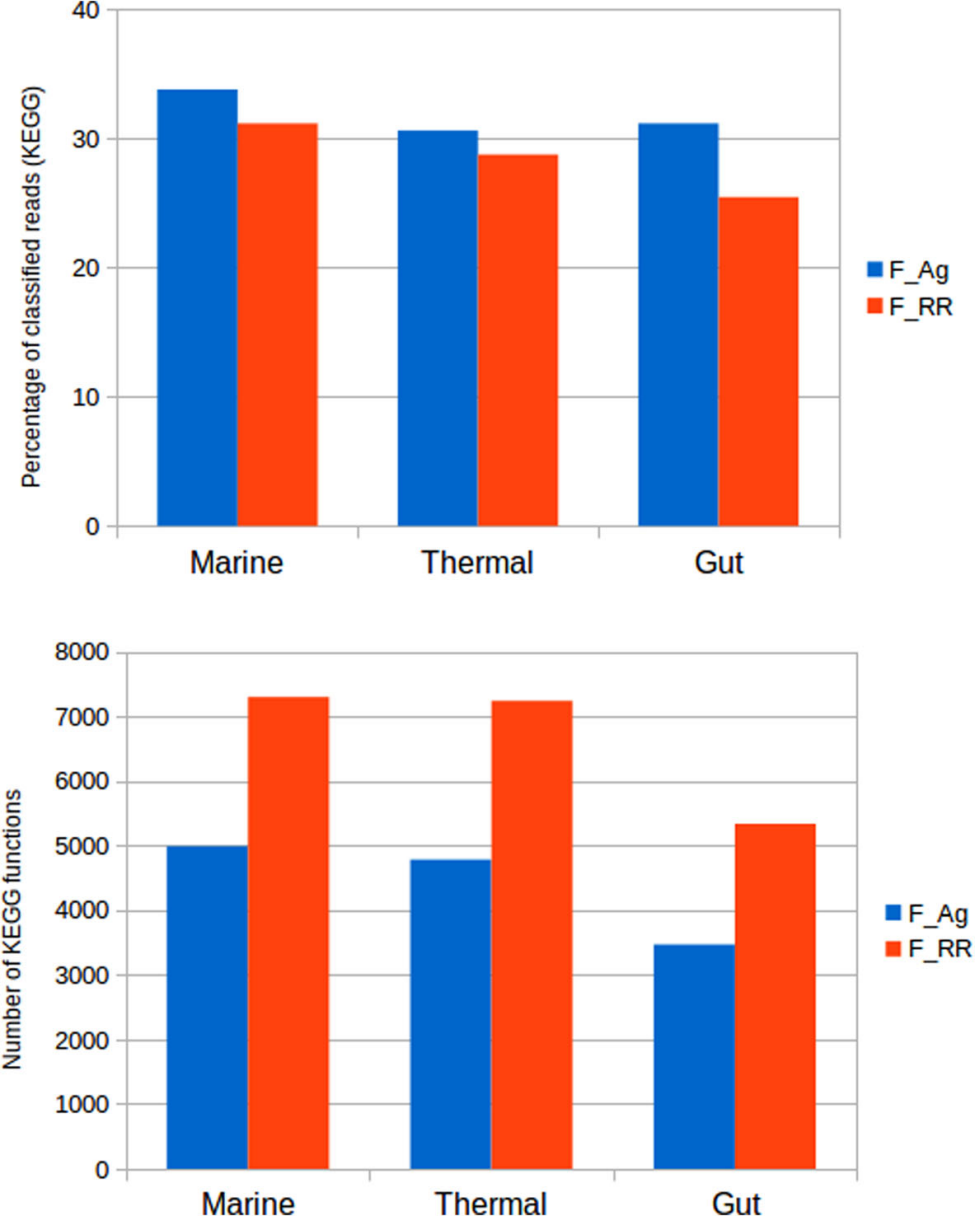
Functional classification of real metagenomes. Top, percentage of reads classified in KEGG functions, by raw reads and assembly approaches, in the three metagenomes. Bottom, number of KEGG functions

We studied the distribution of functions by the assignment of reads to KEGG functions with the Ag (F_Ag) and RR (F_RR) approaches. F_Ag is again able to classify more reads than the F_RR in all metagenomes, even if the difference is small (Fig. 9 top). In contrast, F_RR assignment detects a much higher number of KEGGs in all cases (Fig. 9 bottom). These correspond to low-abundance functions. The percentage of functions represented by less than 10 reads in F_RR is 20, 15 and 23% for marine, thermal and gut metagenomes, respectively. These could correspond to low-coverage parts of the metagenome that could not be assembled. Also, these could correspond to false positives in F_RR annotation, as described when working with mock communities.

**Fig. 10:**
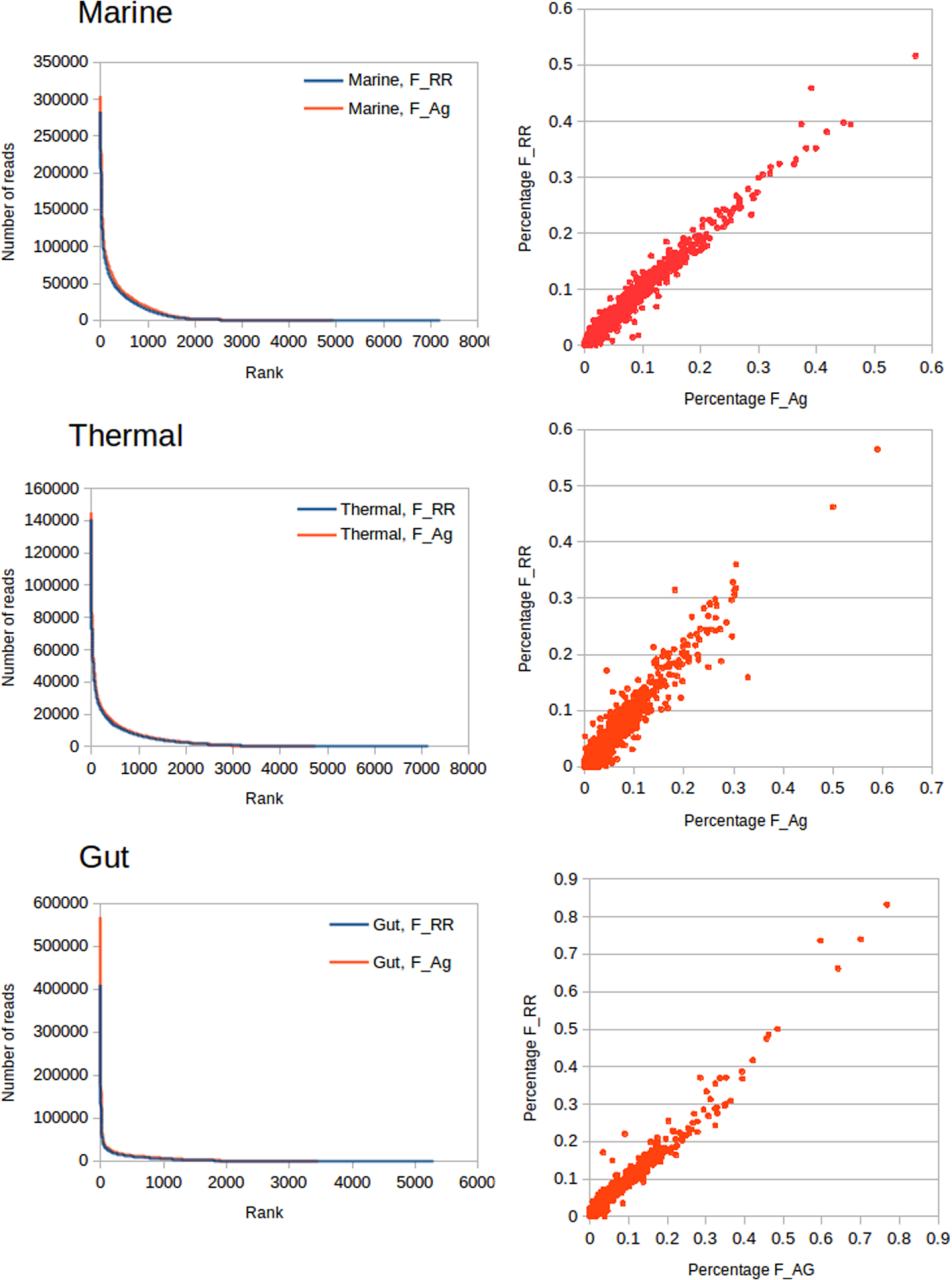
Comparison of functional assignments for all real metagenomes. Left: Rank/abundance curves for the distribution of KEGG functions the three metagenomes, classifying either by raw reads (F_RR) or by assembly and mapping to genes (F_Ag). Right: Scatter plots showing the abundance percentages for each KEGG function for both approaches

Figure 10 (left) shows the distribution of abundances of each KEGG function as rank-abundance curves. Distributions for F_Ag and F_RR are almost indistinguishable, except for the higher number of KEGG functions predicted by F_RR, and the slightly higher abundance for all functions using the assembly, because of the higher number of reads assigned by this method. A comparison of the abundance of KEGG functions can be seen in Fig. 10 (right), where the good fit indicates that there are not big differences between the functional assignments by both methods. The number of discordant assignments (reads classified to different functions by both methods) is low: 1.49, 2.21 and 0.88% for marine, thermal and gut metagenomes, respectively.

## Discussion

Different approaches can be used for the taxonomic and functional annotation of metagenomes. Working with raw reads, taxonomic annotation can be done using homology, k-mer composition or gene marker searches. But we also can assemble the reads and use the assembly as a framework for the annotation, since this will provide longer fragments of genes (or complete ones) and contextual information. There is not a standard way of proceed, and examples of each approach can be found in the literature. However, it is unclear how the diverse approaches can influence the accuracy of the results. We wanted to explore the characteristics of each method to, if possible, provide hints helping the choice of the most appropriate approach.

Regarding taxonomic annotation, our study shows that the differences between different methods are significant (Additional file 7: Figure S7). Especially Kraken2 and Metaphlan2 produce taxonomic assignations that are quite different to the ones obtained using assembly-based or raw read assignment approaches.

Assembly and especially the assignment of raw reads are demanding methods in time and computational resources. In contrast, Kraken2 and Metaphlan2 are very fast methods that can be very useful to obtain a quick view of the diversity of the metagenomes. Nevertheless, the analysis of mock metagenomes shows that these methods are less accurate, especially for non-human-associated environments. They are rather sensitive to the composition of the databases, and their performance decreases when rare species are present in the metagenome. This drawback also affects to the assignment of raw reads by homology.

While for the methods based on annotation of reads database completeness is the main factor determining their performance, for the assembly approaches the critical issue is metagenomic coverage, that in turn influences the completeness of the assembly. When the assemblies recruit 85% or more of the original reads, assembly approaches are more advantageous in terms to percentage of reads classified, smaller number of errors and importantly, similarity to the real scenario. When the coverage is reduced because of both a high microbial diversity and a small number of reads, the assignment of raw reads could be preferred. Assembly approaches seem to be less influenced than others by database completeness because having longer sequences (full genes instead of short reads) is advantageous when only distant homologies can be found, and, for taxonomic annotation, having the contextual information of the contigs helps to infer annotations for all genes on it. Nevertheless, they are also affected to some extent by database composition.

Therefore, when dealing with small metagenomes from well-studied ecosystems, such as these human-associated, the usage of raw read assignment can be preferred for taxonomic assignments, at least for the ranks considered in this study. In other instances, assembly approaches should be favoured. This is especially true if we want to obtain bins, where co-assembly of metagenomes is mandatory. We did not consider the effect of co-assembly in the taxonomic annotation, but since it helps to obtain more and longer contigs and therefore to map more reads to the assembly, it is expected to improve the annotations even more. It would be also possible to follow a combined approach in which assembly is done and used as a reference, and then the remaining unmapped reads are classified independently.

For functions, the KEGG assignments for the real metagenomes show a high degree of correlation between assembly and raw read annotation. Short reads are often not discriminative enough to distinguish between functions, and consequently assembly annotation provides a higher percentage of functional classification. On the other hand, functions represented by a few reads will be probably missed by the assembly approaches. Because of this, raw read assignment provides a higher number of functions than the assembly. Given these advantages and disadvantages of each method, if one is interested in looking for specific functions, a combination of both approaches would be advisable.

## Conclusions

When choosing the most appropriate method for analysing metagenomes, several factors must be taken into account: the underlying diversity and richness, often related to the type of habitat, will influence coverage because rich and diverse samples are more difficult to assemble. Also, metagenomes from less studied habitats will likely find less similarities in the databases. Therefore, when dealing with small metagenomes from well-studied ecosystems, the usage of raw read assignment can be advantageous. Otherwise assembly approaches are more accurate.

## Methods

We have used three different metagenomes: 1) a microbial mat metagenome from a hot spring in Huinay (Chile), corresponding to a sample taken at 48 °C, and sequenced using Illumina HiSeq (82.7 M reads, 9.8 G bases, accession SRP104009) [24] 2) A marine metagenome corresponding to Malaspina sampling expedition, taken at 3 m depth in the Pacific Ocean [25], also sequenced with Illumina HiSeq (168.9 M reads, 16 G bases). 3) A gut metagenome from the human microbiome project [26], sequenced with the Illumina Genome Analyzer II (68.1 M reads, 6.4 G bases, accession SRS052697).

For assessing the performance of the approaches, we used mock metagenomes of different sizes (0.2 M, 0.5 M, 1 M, 2 M, 5 M reads) built with genomes of species significantly associated to each of the three environments considered: marine, thermal and gut. We calculated the associations between species and environments as in [20]. We selected sets of 35 environment- associated species with complete genomes available (Additional file 8: Table S1), and calculated their abundance ratios following a hypergeometric distribution, used to simulate the ratios of species in samples [28]. Knowing these ratios and the total number of reads, we estimated how many reads of each species must be taken and created the mock metagenome by simulating the sequencing of the required number of paired-end reads from these genomes.

For analysing the mock metagenomes, we followed the same approaches above, but we removed the corresponding genomes in the NR database used for homology searching. We also created custom databases for Kraken2 and Metaphlan2 in which we also removed these genomes.

A schematic description of the procedure of analysis can be seen in Fig. 1. The taxonomic classification of the raw reads (RR) was obtained by direct homology searches against GenBank NR database (release 223, December 2017) using DIAMOND (v0.9.13.114) with a minimum e-value threshold of 1e-03 [19]. The taxonomic annotations were done using a last-common ancestor (LCA) algorithm. LCA first select the hits having at least 80% of the bitscore of the best hit and overcoming the minimum identity threshold set for a particular taxonomic rank (85, 60, 55, 50, 46, 42 and 40% for species, genus, family, order, class, phylum and superkingdom ranks, respectively) [29]. This means that in order to classify a sequence at the phylum taxonomic rank, for instance, hits for that sequence must be at least 42% identical. Then it looks for the common taxon for all hits at the desired taxonomic rank (although some flexibility is allowed, for instance admitting one outlier if the number of hits is high). In case that a common taxon is not found, the read is unassigned. For the functional annotation of the raw reads (F_RR), KEGG [30] was used as the reference functional classification, and the reads were annotated using the best hit to this KEGG database.

The set of reads was also assembled and annotated. We used the SqueezeMeta pipeline [21] for this task, that automatizes all steps of metagenomic analysis. The assembly was done using Megahit (v1.1.2) [18], followed by gene prediction using Prodigal (v2.6.2) [22]. The predicted genes were searched for homologies against GenBank NR and KEGG databases using DIAMOND, and processed with the LCA algorithm, as above. This produces taxonomic and functional assignments for the genes in the contigs.

A taxonomic classification for the whole contig can be obtained as the consensus of the annotations of its genes. The criteria for declaring a consensus taxon are: 50% of all genes and 80% of the annotated genes in the contig must belong to the taxon (some genes may not have annotation). Otherwise, the contig is left unassigned. This approach has the advantage of allowing the annotation of many additional genes (those in the contig that were not classified directly, including orphans), but it has the drawback of dropping the original annotations for the genes if a consensus is not found. Notice that under these criteria, short contigs comprising just one gene get the annotation of their only gene.

Once genes and contigs are annotated, we classified the reads mapping them against the contigs using Bowtie2 (v2.2.6) [31], and inheriting the annotation of the corresponding gene or contig. Also, we investigated a combined approach merging these two strategies, in which reads first inherit the annotation of the contig, and then the one of the gene if the contig did not provide an annotation. These approaches will be referred as annotation by assembly/genes (Ag), assembly/contigs (Ac), and assembly/combined (Am). For functional classification, only mapping against genes was used (F_Ag), since there is not contig annotation for functions (each gene has a different function).

We also used two other approaches widely used in metagenomic analysis for taxonomic assignment. First, assignment by means of k-mer composition using Kraken2 (KR) [12]. Second, the clade-specific gene marker searching of Metaphlan2 (MP) [14]. These methods are not suitable for functional assignment.

For analysing the mock metagenomes, we followed these same approaches, but we removed the corresponding genomes in the NR database used for homology searching. We also created custom databases for Kraken2 and Metaphlan2 in which we removed these genomes as well.

For each metagenome, we compiled tables with the taxonomic or functional assignment of each of the reads by all methods. These tables were used to calculate the functional and taxonomic profiles that were used in the comparison.

Such [20, 28] comparison between taxonomic profiles for the different methods (and to real values in case of mock communities) was done by calculating the Bray-Curtis dissimilarity measure between them. Comparison between functional profiles was done measuring the percentage of reads with divergent annotations between them.

The datasets used and/or analyzed during the current study available from the corresponding author on reasonable request.

## Supplementary information

**Additional file 1: Figure S1.** Taxonomic assignments for the mock communities, at the family rank. Ac, Megahit assembly and mapping reads to contigs. Ag, Same but mapping reads to genes. Am, same but mapping genes first to contigs, then to genes. RR, raw reads assignment. KR: Kraken. MP: Metaphlan2. Left: All reads considered. Right: Discounting unclassified reads. Numbers above the bars in the right panels correspond to the Bray-Curtis dissimilarity to the composition of the original microbiome.

**Additional file 2: Figure S2.** Bray-Curtis dissimilarity between assignment methods for mock metagenomes. The “real” column indicated the distance to the real composition of the mock metagenome.

**Additional file 3: Figure S3.** Bray-Curtis dissimilarity between assignment methods by assembly, comparing Megahit and metaSPAdes assemblers. Left: mock communities. Right: real metagenomes.

**Additional file 4: Figure S4.** Bray-Curtis dissimilarity to the real composition of the mock community, at family taxonomic rank. For several sample sizes, at phylum rank. Ac, Assembly and mapping reads to contigs. Ag, Same but mapping reads to genes. Am, same but mapping genes first to contigs, then to genes. RR, raw reads assignment. KR: Kraken2. MP: Metaphlan2.

**Additional file 5: Figure S5.** Taxonomic assignments for the real communities, at the family rank. Ac, Megahit assembly and mapping reads to contigs. Ag, Same but mapping reads to genes. Am, same but mapping genes first to contigs, then to genes. RR, raw reads assignment. KR: Kraken. MP: Metaphlan2. Left: All reads considered. Right: Discounting unclassified reads.

**Additional file 6: Figure S6.** Bray-Curtis dissimilarity between assignment methods for real metagenomes.

**Additional file 7: Figure S7.** Significance of differences for taxonomic assignment methods*.* For each analysis method (Ac, Ag, Am, RR, KR, MP), the left-side boxplot shows the Bray-Curtis dissimilarities between the taxonomic profile (phylum level) obtained with that method and the taxonomic profiles obtained with the rest of the methods. This was done separately for the three real metagenomes and the three mock metagenomes with one million reads. The right side boxplot shows the pairwise Bray-Curtis dissimilarities between the taxonomic profiles (phylum level) obtained with the rest of the methods. Significant differences (Kruskal-Wallis, *p* < 0.05) are denoted with a red asterisk.

**Additional file 8: Table S1.** Abundances and PATRIC accession numbers [32] of different species in the mock metagenomes.

**Additional file 9: Table S2.** Percentage of divergent assignments for mock communities. “Real” indicates the differences with the real functional composition of the metagenome.

## Data Availability

All datasets used and/or analyzed during the current study available from the corresponding author on reasonable request. Metagenomic datasets can be found in the SRA archive with accession SRP104009 (Thermal dataset), SRS052697 (Gut dataset).
