# Supplementary figures and images for: Assessing the performance of different approaches for functional and taxonomic annotation of metagenomes
Source: BMC Genomics. 2019 Dec 10;20:960. doi: 10.1186/s12864-019-6289-6 (PMC6902526; doi:10.1186/s12864-019-6289-6)

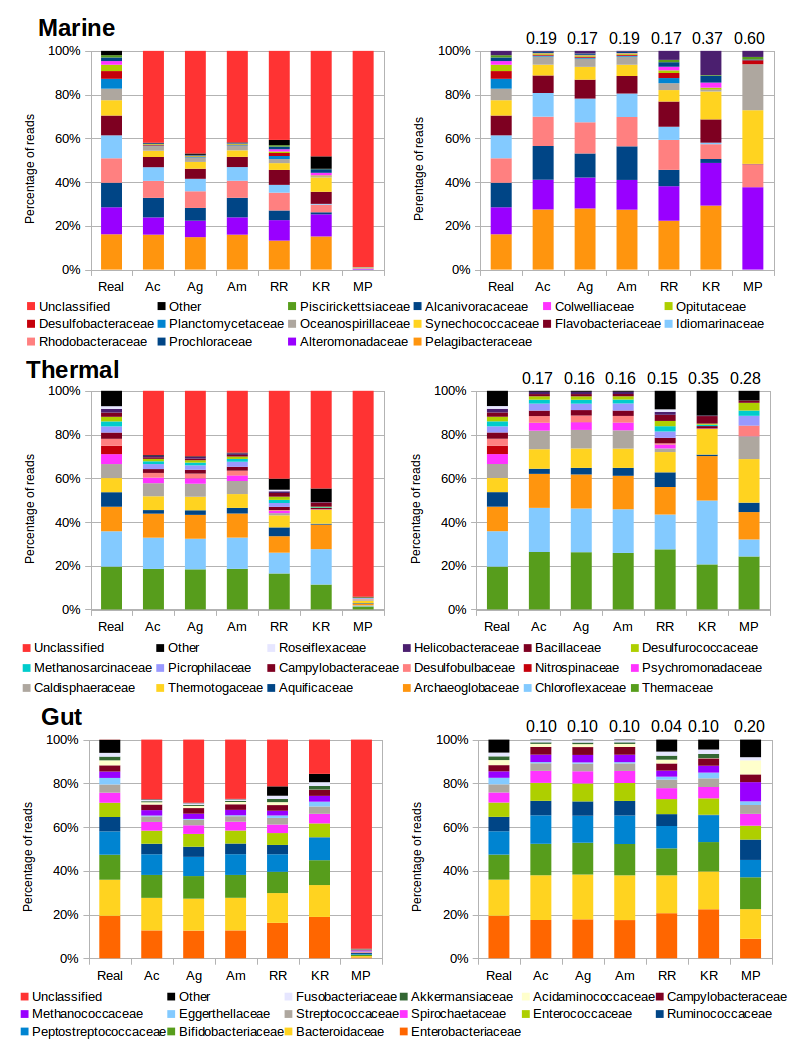

Supplement: Supplementary file 1 — Additional file 1: Figure S1. Taxonomic assignments for the mock communities, at the family rank. Ac, Megahit assembly and mapping reads to contigs. Ag, Same but mapping reads to genes. Am, same but mapping genes first to contigs, then to genes. RR, raw reads assignment. KR: Kraken. MP: Metaphlan2. Left: All reads considered. Right: Discounting unclassified reads. Numbers above the bars in the right panels correspond to the Bray-Curtis dissimilarity to the composition of the original microbiome. [file 12864_2019_6289_MOESM1_ESM.png]

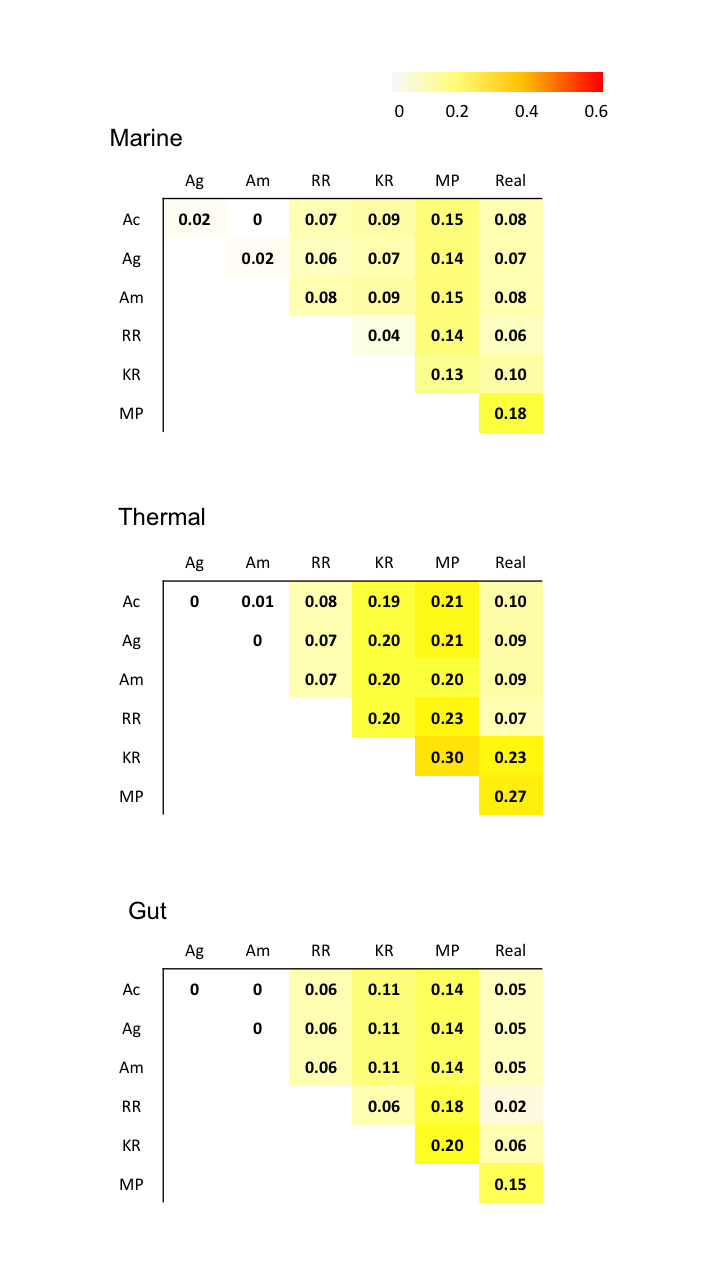

Supplement: Supplementary file 2 — Additional file 2: Figure S2. Bray-Curtis dissimilarity between assignment methods for mock metagenomes. The “real” column indicated the distance to the real composition of the mock metagenome. [file 12864_2019_6289_MOESM2_ESM.png]

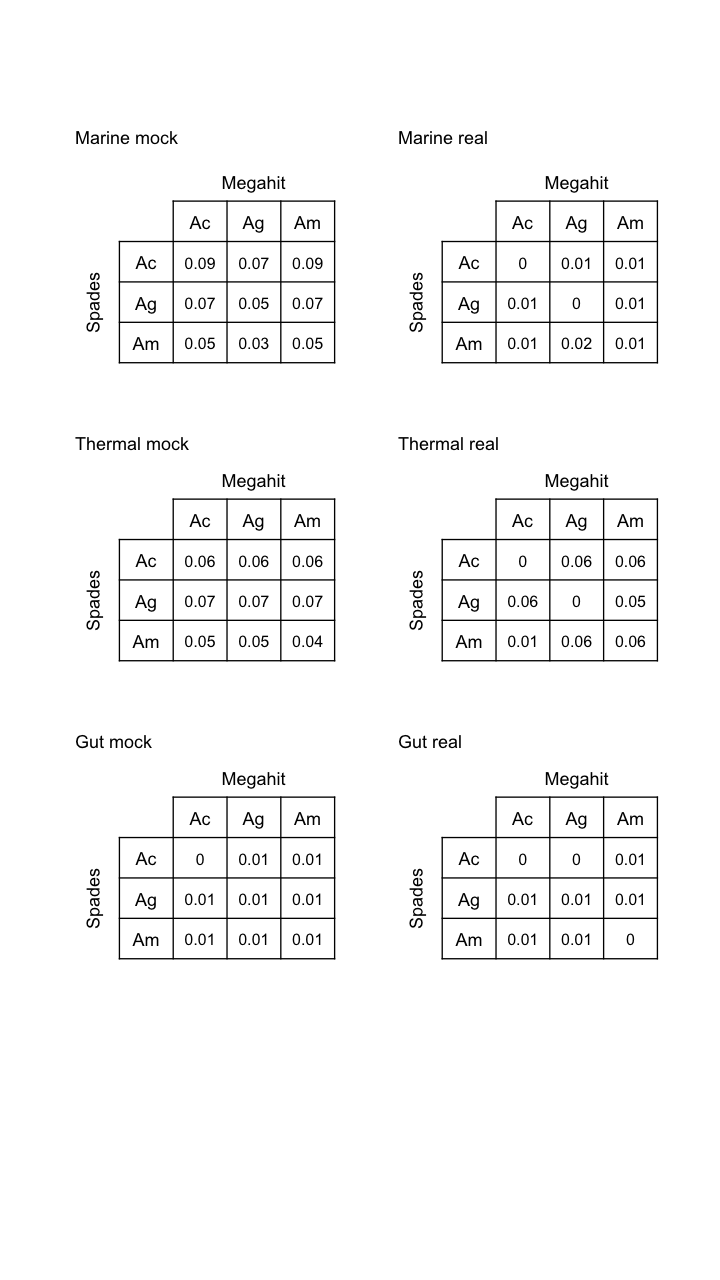

Supplement: Supplementary file 3 — Additional file 3: Figure S3. Bray-Curtis dissimilarity between assignment methods by assembly, comparing Megahit and metaSPAdes assemblers. Left: mock communities. Right: real metagenomes. [file 12864_2019_6289_MOESM3_ESM.png]

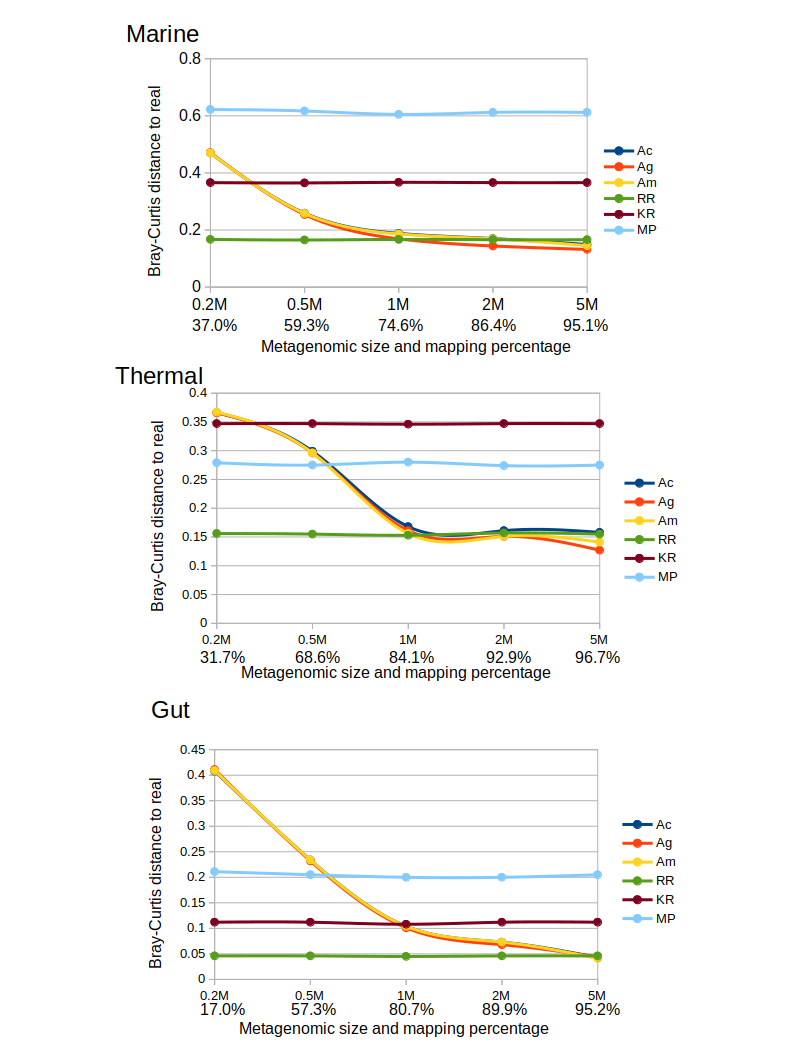

Supplement: Supplementary file 4 — Additional file 4: Figure S4. Bray-Curtis dissimilarity to the real composition of the mock community, at family taxonomic rank. For several sample sizes, at phylum rank. Ac, Assembly and mapping reads to contigs. Ag, Same but mapping reads to genes. Am, same but mapping genes first to contigs, then to genes. RR, raw reads assignment. KR: Kraken2. MP: Metaphlan2. [file 12864_2019_6289_MOESM4_ESM.png]

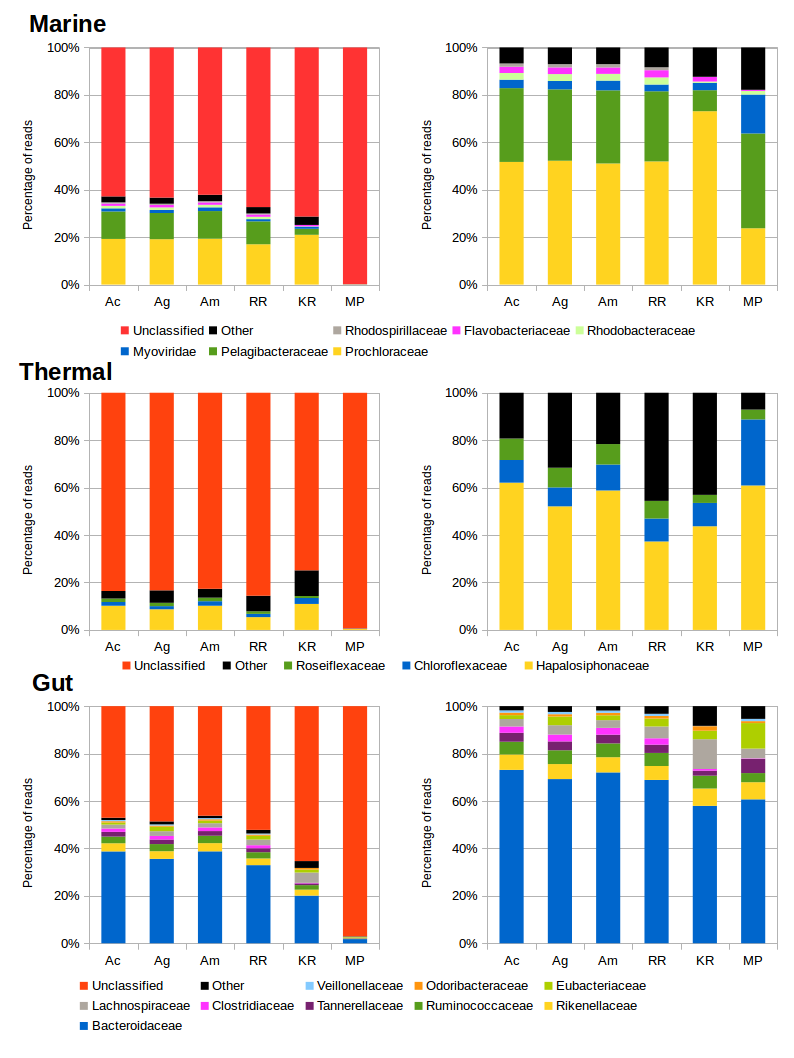

Supplement: Supplementary file 5 — Additional file 5: Figure S5. Taxonomic assignments for the real communities, at the family rank. Ac, Megahit assembly and mapping reads to contigs. Ag, Same but mapping reads to genes. Am, same but mapping genes first to contigs, then to genes. RR, raw reads assignment. KR: Kraken. MP: Metaphlan2. Left: All reads considered. Right: Discounting unclassified reads. [file 12864_2019_6289_MOESM5_ESM.png]

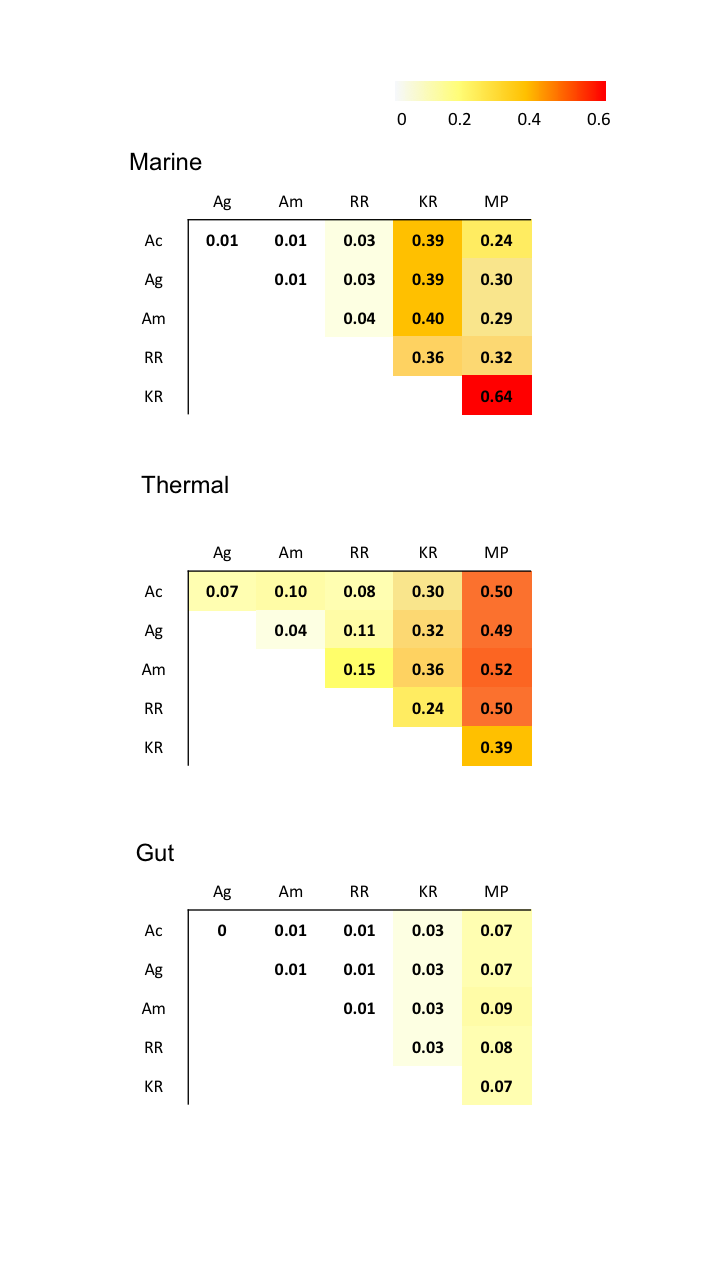

Supplement: Supplementary file 6 — Additional file 6: Figure S6. Bray-Curtis dissimilarity between assignment methods for real metagenomes. [file 12864_2019_6289_MOESM6_ESM.png]

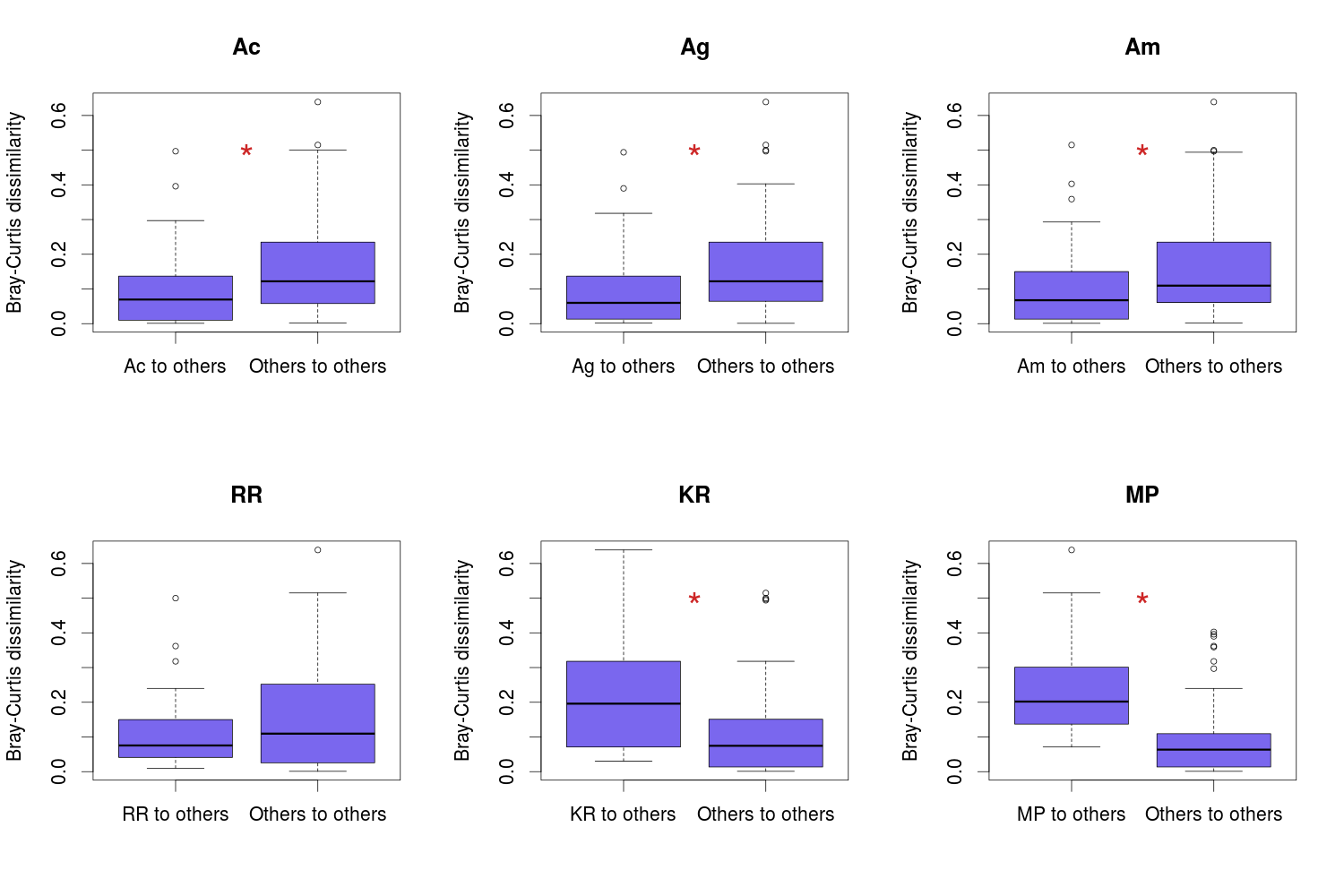

Supplement: Supplementary file 7 — Additional file 7: Figure S7. Significance of differences for taxonomic assignment methods. For each analysis method (Ac, Ag, Am, RR, KR, MP), the left-side boxplot shows the Bray-Curtis dissimilarities between the taxonomic profile (phylum level) obtained with that method and the taxonomic profiles obtained with the rest of the methods. This was done separately for the three real metagenomes and the three mock metagenomes with one million reads. The right side boxplot shows the pairwise Bray-Curtis dissimilarities between the taxonomic profiles (phylum level) obtained with the rest of the methods. Significant differences (Kruskal-Wallis, p < 0.05) are denoted with a red asterisk. [file 12864_2019_6289_MOESM7_ESM.png]
