# Supplementary material for: Assessing the performance of different approaches for functional and taxonomic annotation of metagenomes
Source: BMC Genomics. 2019 Dec 10;20:960. doi: 10.1186/s12864-019-6289-6 (PMC6902526; doi:10.1186/s12864-019-6289-6)
Supplement: Supplementary file 9 — Additional file 9: Table S2. Percentage of divergent assignments for mock communities. “Real” indicates the differences with the real functional composition of the metagenome. [file 12864_2019_6289_MOESM9_ESM.docx]

| Marine | F_RR | Real |
| --- | --- | --- |
| F_Ag | 1.77 | 3.51 |
| F_RR |  | 2.53 |

| Thermal | F_RR | Real |
| --- | --- | --- |
| F_Ag | 2.74 | 5.39 |
| F_RR |  | 4.24 |

| Gut | F_RR | Real |
| --- | --- | --- |
| F_Ag | 1.84 | 3.46 |
| F_RR |  | 2.45 |

Table S2: Percentage of divergent assignments for mock communities. "Real" indicates the differences with the real functional composition of the metagenome
